# Children With Reading Difficulty Rely on Unimodal Neural Processing for Phonemic Awareness

**DOI:** 10.3389/fnhum.2019.00390

**Published:** 2019-11-14

**Authors:** Melissa Randazzo, Emma B. Greenspon, James R. Booth, Chris McNorgan

**Affiliations:** ^1^Department of Communication Sciences and Disorders, Adelphi University, Garden City, NY, United States; ^2^Department of Psychology, State University at Buffalo, New York, NY, United States; ^3^Department of Psychology, Monmouth University, New Jersey, NJ, United States; ^4^Department of Psychology and Human Development, Vanderbilt University, Tennessee, TN, United States

**Keywords:** reading difficulty, crossmodal integration, phonemic awareness, audiovisual integration, fMRI—functional magnetic resonance imaging, dyslexia

## Abstract

Phonological awareness skills in children with reading difficulty (RD) may reflect impaired automatic integration of orthographic and phonological representations. However, little is known about the underlying neural mechanisms involved in phonological awareness for children with RD. Eighteen children with RD, ages 9–13, participated in a functional magnetic resonance imaging (fMRI) study designed to assess the relationship of two constructs of phonological awareness, phoneme synthesis, and phoneme analysis, with crossmodal rhyme judgment. Participants completed a rhyme judgment task presented in two modality conditions; unimodal auditory only and crossmodal audiovisual. Measures of phonological awareness were correlated with unimodal, but not crossmodal, lexical processing. Moreover, these relationships were found only in unisensory brain regions, and not in multisensory brain areas. The results of this study suggest that children with RD rely on unimodal representations and unisensory brain areas, and provide insight into the role of phonemic awareness in mapping between auditory and visual modalities during literacy acquisition.

## Introduction

Phonological awareness skills are important in learning to read in alphabetic languages (Bus and van IJzendoorn, 1999; Ehri et al., 2001; Lonigan et al., 2009). Phonemic awareness skills are metalinguistic skills that include the ability to manipulate the sound structure of oral language (Wagner and Torgesen, 1987; Share, 1995; Ehri et al., 2001; Ziegler and Goswami, 2005). These skills are a subset of phonological awareness skills that operate on smaller phonological segments at the level of the individual speech sound, or phoneme (Anthony et al., 2003; Ziegler and Goswami, 2005). Phonemic awareness is strongly associated with word reading and is more highly correlated with reading skills than rhyme-level awareness (Melby-Lervåg et al., 2012). Phonemic awareness, however, can be further subdivided into two complementary skills: synthesis and analysis (Perfetti et al., 1987). Synthesis refers to the ability to combine isolated phonemes into syllables or words. For example, blending phonemes /k/, /æ/, and /t/ into the word “cat” demonstrates the synthesis principle. Analysis refers to the ability to break words or syllables into smaller speech segments. For example, in elision tasks, given the instruction to produce “cat” without the “/k/,” the verbal response “/æt/,” demonstrates the analysis principle.

### Component Constructs of Phonological Awareness

Synthesis and analysis are often considered a unified construct in studies examining the development of phonological awareness (Anthony and Lonigan, 2004; Lonigan et al., 2009). Several studies, however, suggest that these two skills are distinct aspects of phonemic awareness that are performed by divergent manipulations. Phoneme synthesis and analysis skills appear to have different developmental trajectories and play different roles in the reading acquisition process. Phoneme synthesis, which is the phonemic awareness skill underlying blending tasks, develops before phoneme analysis, which is the phonemic awareness skill underlying tasks requiring segmentation (Anthony et al., 2003; Lonigan et al., 2009). Different developmental trajectories suggest there is at least a partial dissociation between the contributions of these two skills during reading development. Studies examining the relationship between phoneme synthesis and analysis skills with early reading development and instructional outcomes have reinforced the notion that these constructs differentially contribute to reading performance. For example, in their study of preschoolers prior to formal reading instruction, Burgess and Lonigan (1998) found that letter-sound knowledge was a unique predictor of phoneme analysis in elision tasks but not of synthesis in blending tasks. Similarly, Kroese et al. (2000) found that elision along with phoneme reversal were strongest predictors of reading and spelling ability in children in late elementary school, with blending predicting less of the variance. These findings support both the arguments that analysis and synthesis are constructs that tap different aspects of phonemic awareness, and that a bidirectional relationship exists between phonemic awareness and reading skill.

An earlier attempt to more precisely characterize this relationship was carried out by Perfetti et al. (1987), who examined the unique contributions of synthesis and analysis skills towards reading development between first and second grade. This longitudinal study followed first graders exposed to either of two types of reading instruction emphasizing direct (i.e., phonics) or indirect (i.e., whole-word) decoding. The study demonstrated that the relationship between these skills and reading ability depended on the type of reading instruction administered, suggesting that these two phonemic awareness skills are differentially related to different reading strategies, and thus represent distinct skills. The authors concluded that synthesis skills enable reading development, presumably through bootstrapping orthographic assembly from existing skill in phonological assembly. However, acquired reading skill enables later analysis skills, thus providing an account in which phonemic awareness skills and reading development are mutually dependent.

A prominent model of reading acquisition has argued that phonemic-level awareness is a result of increased sensitivity to phonemes by exposure to orthography (Ziegler and Goswami, 2005), and consequently plays a crucial role in bridging phonological representation to orthographic input. This idea is consistent with the findings described above showing a bidirectional relationship between phoneme analysis skills and typically-developing reading ability, and is supported by numerous studies showing that orthographic knowledge influences phonological processing (Ehri and Wilce, 1980; Stuart, 1990; Castles et al., 2003; Desroches et al., 2010). Upon acquiring letter knowledge, readers may utilize this information on phoneme judgment tasks. Given even relatively brief exposure to orthographic representations, preschoolers also demonstrate a similar influence of orthography on phonemic awareness tasks (Castles et al., 2011). This suggests that a crossmodal influence of orthographic and phonological representations may accompany the learning of the alphabetic principle, and continue as a child learns to read. This is consistent with the argument that phoneme analysis skills are an experience-dependent outcome of skilled reading in opaque orthography (Mann and Wimmer, 2002).

### Crossmodal Processing and Phonemic Awareness

Interventions for phonological awareness skills are typically motivated by the assumption that reading difficulty (RD) arises from deficits in auditory processing or phonological representation (Tallal, 1980; Shaywitz and Shaywitz, 2005). However, the role of orthography in the development of phonemic awareness skills and the reciprocal nature of phonemic awareness and reading ability suggest that a failure to integrate letters and speech sounds may contribute substantially towards RD. It has been suggested that decreased phonological awareness in children with RD may reflect impaired automatic integration of orthographic and phonological representations, signifying a crossmodal deficit in integrating visual letters and auditory speech sounds, rather than a unimodal auditory processing deficit (Vaessen et al., 2009). Deficits in letter-sound integration may be attributed to decreased automatization of pairing these associations (Bakos et al., 2017). Multisensory interactions provide useful constraints on lexical activations in either modality to the extent that they help disambiguate multiple competing representations (Seidenberg and McClelland, 1989; Harm and Seidenberg, 1999). Moreover, training such mapping between modalities, in turn, drives visual specialization (Fraga González et al., 2017) and can improve reading fluency (Žarić et al., 2015). We take the well-supported position that developmental reading difficulties arise in large part from an inability to accurately and quickly map between phonological and orthographic representations, or between auditory and visual modalities (Booth et al., 2004, 2007; Cao et al., 2006; Bitan et al., 2007).

Because they identify the neural correlates of cognitive processing, neuroimaging, and neurophysiological studies have contributed much towards our understanding of the interacting systems involved in typical and disordered reading. Neuroimaging and neurophysiological studies examining audiovisual integration of letters and speech sounds in children suggest that RD may be partly attributable to difficulties in crossmodal integration of these entities. In this view, crossmodal associations between the visual letter and auditory speech sound are reinforced through reading experience, which then refines phonemic awareness skills in typically developing (TD) readers (Blau et al., 2009). Blau et al. (2010) demonstrated enhanced letter-sound integration in audiovisual conditions for TD compared to dyslexic readers in a series of functional magnetic resonance imaging (fMRI) studies. These studies collectively identified an audiovisual integration network that includes brain regions actively engaged in both unimodal and crossmodal processing of phonological and orthographic representations.

Phonological processing and representation are critically supported by the primary auditory cortex (or Heschl’s gyrus) and superior temporal gyrus (STG; Hickok and Poeppel, 2000; Humphries et al., 2014). Though coarsely characterized as a unimodal region, a posterior sub-region in the STG called the Planum Temporale (PT) and the posterior superior temporal sulcus (pSTS), both of which are anatomically proximal to visuomotor processing pathways, have been implicated as audiovisual integration sites in multiple domains (Calvert, 2001; Beauchamp et al., 2004; Stevenson and James, 2009; van Atteveldt et al., 2009). Consistent with a model in which RD arises in part from disordered audiovisual integration, crossmodal activation within PT and pSTS has been shown to differentiate TD readers and children with RD (Blau et al., 2010). In this neuroanatomical model of audiovisual letter to sound mapping, orthographic representations mediate the auditory cortical response to speech sounds in the PT only for TD readers. RD readers, in contrast, under-activate unisensory processing regions to speech sounds and visual letters in the STG and fusiform gyrus (FG), respectively, possibly resulting from deficient crossmodal mediation. Consistent with this model, Blau and colleagues found that the visual response to print in the FG was associated with crossmodal processing effects in the PT, mediated by reading skill. Price and Devlin’s (2011) Interactive Account similarly argues that audiovisual integration plays a critical mediating role in reading development, claiming that the specialization of FG for orthographic processing is a consequence of internally-driven (i.e., top-down) phonological input facilitating the perceptually-driven (i.e., bottom-up) visual object processing system. This facilitation hinges on effective crossmodal integration. The introduction of additional disambiguating information helps reduce uncertainty and identifies the most probable lexical representation. It follows that ineffective crossmodal integration may provide no useful information, or even misleading information. Uncovering and improving how children with RD cope with this challenge is thus a central goal for those who research and work with these populations. Collectively, this body of literature suggests that typical reading development relies on successful audiovisual integration and that RD is associated with reduced integration between modalities (Richlan, 2019).

### How and When do Children With Reading Difficulty Make Use of Phonemic Awareness?

Given the body of research implicating disrupted audiovisual integration in RD, and that remediation for poor readers is often focused on assessment and improvement of phonemic awareness, it is important to understand the neural mechanisms underlying distinct aspects of phonemic awareness, and how they interact with those underlying audiovisual integration. Little is known about the underlying neural mechanisms involved in phonological awareness for children with RD in deep orthographies; indeed, rectifying this gap in the literature partly motivates the present research.

Frost et al. (2009) examined the relationship between phonemic awareness and brain activation for print and speech in TD readers and found that a composite measure of higher phonemic awareness skill was associated with increased activation in the left STG for the processing of print. Although the study examined print and speech processing in separate modality conditions, phoneme analysis was more sensitive to the overlap of print and speech processing than phoneme synthesis. This suggests that phoneme analysis skills are more related to the audiovisual integration of speech and print processing than are phoneme synthesis skills.

The position that normal and disordered reading differentially engages audiovisual integration processes is supported by an fMRI study, which found that the connection between audiovisual integration and reading skill differed for TD and RD readers (McNorgan et al., 2013). This study showed that Elision skill was related to neural activity when engaged in audiovisual processing, but not auditory- or visual-only processing. Moreover, this relationship held in TD but not children with RD, even though TD and RD groups had overlapping Elision scores. This relationship between phoneme analysis and audiovisual lexical processing was driven by sensitivity to orthographic congruency in the FG and pSTS, regions strongly associated with orthographic processing (Tsapkini and Rapp, 2010) and audiovisual integration (Calvert, 2001), respectively. Similarly, Gullick and Booth (2014) found that pSTS activity is related to functional connectivity in the arcuate fasciculus, a tract that is related to individual differences in reading skill, during crossmodal rhyme judgment in typical readers. Broader consideration of the body of work on these regions comprising a crossmodal reading network suggests that phoneme analysis is related to audiovisual integration processes in TD children and that RD is associated with a breakdown in this relationship.

The purpose of the current study is to examine how phonemic awareness supports online rhyme judgment, a phonologically-based lexical task, in children with RD. The null effects associated with their RD sample necessarily lead McNorgan et al. (2013) to conclude there was no evidence of any relationship between neural processing dynamics and phonemic awareness skills in their RD sample. This consequently limited their framing of RD to the neurotypical processes in which they do not engage during reading, leaving a gap in our understanding of the neural correlates of phonological processing during reading in children with RD. It remains unclear which phonological processes children with RD do engage while reading, and how this processing relates to RD severity. We take advantage of our experimental design to examine how constructs of phonemic awareness are related to the degree of impairment and the magnitude of brain activity under different modality conditions. As phoneme analysis involves advancing abilities over time, we explored the influence of modality in rhyme judgment in children ages 9–13 years old who have received several years of reading instruction and span a continuum of reading ability.

We focused our analysis on a sub-network of left hemisphere regions for which the neuroimaging literature has shown consensus as being involved in phonological and orthographic processing and in audiovisual integration, and has been explicitly implicated in the models reviewed above. The inferior parietal lobule (IPL) is implicated in phonological processing and mapping between orthographic and phonological representations (Bitan et al., 2007; Moll et al., 2014). The FG is recognized for its specialization in print processing in skilled readers (Shaywitz et al., 2002; McCandliss et al., 2003; Dehaene and Cohen, 2011). The pSTS is widely regarded as an audiovisual integration site across domains, with a specific role in the integration of letters and speech sounds (Calvert, 2001; van Atteveldt et al., 2009; Blau et al., 2010). Finally, the STG contains associative auditory cortex and plays a critical role in processing phonological word forms (Pugh et al., 2001; Booth et al., 2002; Friederici, 2012).

Investigations of audiovisual integration depend on stimulus congruency, or the correspondence between representations, as this demonstrates how the processing of one representation influences the processing of the other. In studies investigating audiovisual integration in reading, congruency is examined between unimodal and crossmodal presentations at a small grain size (i.e., letters and speech-sounds; Froyen et al., 2008) or lexical rhyme judgment at a larger grain size (McNorgan et al., 2013, 2014). Given the inconsistency of the English orthography at the smaller grain sizes (e.g., letters), large grain sizes (e.g., words, syllables or rimes) play a greater role in early reading development because they provide greater consistency (Ziegler and Goswami, 2005) and are sensitive to skill-related differences in audiovisual lexical processing (Kronschnabel et al., 2014). Fluent reading in English necessitates the processing of larger grain sizes because the processing of smaller grain sizes utilizing a letter-by-letter decoding strategy will only be successful with words that have consistent grapheme to phoneme correspondences. Therefore, we assessed the neural response to inter-stimulus phonological congruency for unimodal (auditory-only) and crossmodal (audiovisual) items at the lexical level in a rhyme judgment task.

The body of research indicating that RD arises from a failure to integrate letters and speech sounds suggests that children with RD might favor unimodal processing of lexical items. Accordingly, we hypothesized phonemic awareness tasks in these children would draw on unimodal processing, rather than crossmodal integration, and thus that these effects would be evident in STG and FG, the two nodes in our reading network most strongly associated with unimodal processing of phonology and orthography, respectively. Moreover, given that previous research suggests differential relationships between phoneme analysis and synthesis skills and reading development, we were also interested in whether these skills bear different relationships to brain activation when analyzed within the context of unimodal (auditory-only) and crossmodal (audio-visual) presentation modalities in children along a continuum of reading ability. Because McNorgan et al. (2013) found Elision scores to be unrelated to neural processing under audio-visual presentation conditions in a sample of children with RD closely matched with a TD sample, we anticipated replicating this finding but hypothesized that phonemic awareness, which is associated with crossmodal processing in TD readers, would be predicted by unimodal processing in our larger sample.

## Materials and Methods

### Participants

Participants were selected from among a larger group involved in a study investigating reading development in children with a range of reading ability. All participants were native English speakers, right-handed, had a normal or corrected-to-normal vision, and had no history of psychiatric illness, neurological disease or attention deficit hyperactivity disorder (ADHD). Study participants were recruited from the Chicago metropolitan area. Informed consent was obtained from participants and their parents. The Institutional Review Board at Northwestern University approved all procedures.

Participants with a prior diagnosis of RD were referred for the study. RD was quantified prior to admission to the study as a standard screening procedure. We evaluated non-verbal IQ using the *Wechsler Abbreviated Scale of Intelligence—Second Edition* (WASI-II; Wechsler, 2011) and reading skill using the Word Identification, Word Attack and Reading Fluency subtests of the *Woodcock-Johnson Tests of Achievement—III* (WJ III; Woodcock et al., 2001) and the Sight Word Efficiency (SWE) and Phonetic Decoding Efficiency (PDE) subtests of the *Test of Word Reading Efficiency* (TOWRE; Torgesen et al., 1999). Eighteen children met our eligibility requirements (11 males; mean age = 11 years, 8 months; range = 9 years, 10 months to 13 years, 11 months). Participants were included in the study if, in addition to presenting with a clinical diagnosis of RD, at least two of the five scaled scores were less than or equal to 95, at least one score at or below 91, and the mean of all scaled scores was less than or equal to 95. Participants had an average of 3.9 out of 5 standard scores below 90. All other scores fell in the average to below-average range across participants. These selection criteria enabled our correlational design to investigate reading skills in otherwise cognitively typical children diagnosed with RD but demonstrating a range of skills. Each participant’s phonemic awareness was measured by the Elision and Blending subtests of the Comprehensive Test of Phonological Processing (CTOPP; Wagner et al., 1999). Scores reflect the number of correct Elision or Blending transformations on a set of 20 progressively more difficult target items. Participants had near-average performance for the Blending and Elision measures of Phonological Awareness (group mean raw score Blending = 9.0, group mean raw score Elision 8.7, test mean raw score = 10). Participants had better than chance performance on the experimental task (*M* = 0.65, *SD* = 0.11) and without evidence of response bias across all scanning sessions, as measured by a d-prime analysis of responses (*M* = 0.64). Summary statistics for these participant characteristics appear in Table 1. We report raw, rather than scaled, CTOPP scores because they were used as dependent measures in the regression analyses that follow.

**Table 1:** Participant characteristics.

Measure	Mean Score (SD)	Range
WASI Performance IQ	100 (13)	74–127
WJ-III Word ID	90 (7)	67–113
WJ-III Word Attack	92 (5)	83–103
WJ-III Reading	90 (10)	67–113
TOWRE SWE	89 (10)	60–113
TOWRE PDE	88 (10)	71–104
CTOPP Elision	8.7 (2.8)	4–12
CTOPP Blending	9.0 (2.2)	5–14

*Note: WASI, Wechsler Abbreviated Scale of Intelligence; WJ-III, Woodcock-Johnson Tests of Achievement—III; TOWRE, Test of Word Reading Efficiency; SWE, Sight Word Efficiency; PDE, Phonetic Decoding Efficiency; CTOPP, Comprehensive Test of Phonological Processing. CTOPP raw scores reported above; CTOPP Elision score max = 20, test mean = 10; CTOPP Blending score max = 20, test mean = 10*.

### Experimental Procedure

#### Rhyme Judgment Task

On each trial, participants were presented with paired stimuli, the order of which was counterbalanced across participants in an event-related paradigm. For each scanning session, stimuli were presented in one of two modality conditions: In the cross-modal auditory/visual (AV) condition, the first item was presented auditorily and the second was presented visually. In the unimodal auditory/auditory (AA) condition, both items were presented in the auditory modality. Half the pairs of stimuli rhymed and half did not, and participants were asked to make a rhyme judgment response by pressing one of two keys on a handheld keypad. Participants were asked to respond as quickly and as accurately as possible, using their right index finger for a yes (rhyme) response and their right middle finger for a no (non-rhyme) response. Participants participated in two runs for each modality condition, each lasting approximately 7 min. Participants generally saw the AV condition followed by the AA condition, though this varied across participants as factors such as task accuracy and movement necessitated reacquiring data.

**Figure 1:**
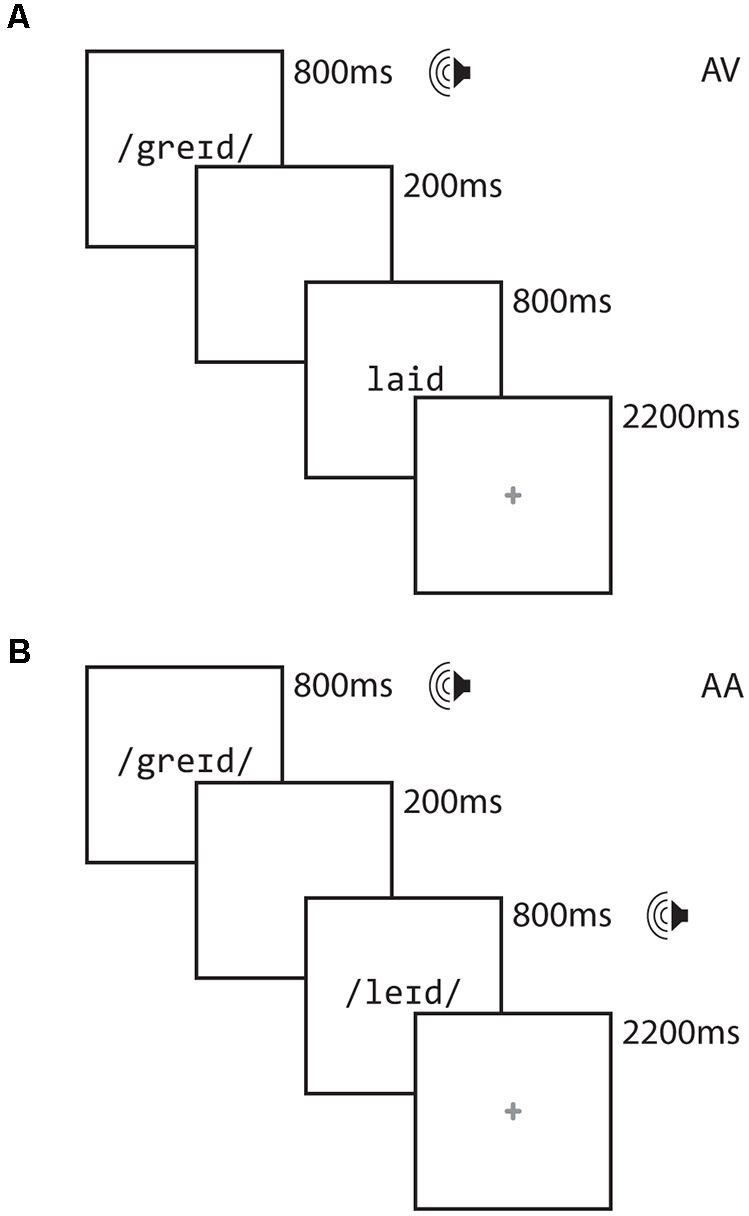
Task diagram for the AV Crossmodal task **(A)** and AA Unimodal task **(B)**.

Figure 1 illustrates the design of each trial. Each stimulus item was presented for 800 ms, separated by a 200 ms interstimulus interval. Participants were free to respond as soon as the second stimulus item was presented. A red cross appeared for 2,200 ms following the presentation of the second word, signaling to the participant to respond if they had not already done so. Responses made after the red cross disappeared from the screen were not recorded and were counted as errors.

We examine rhyme judgment in orthographically consistent (O+) and orthographically inconsistent (O−) conditions, which we crossed with the rhyming (P+) and non-rhyming (P−) lexical conditions. Thus, the lexical trials included two rhyming conditions, comprising orthographically consistent (O+P+ e.g., CAGE RAGE) and orthographically inconsistent (O−P+ e.g., GRADE LAID) pairs. The lexical trials also included two non-rhyming conditions, comprising orthographically consistent (O+P−; e.g., SMART WART) and orthographically inconsistent (O−P−; e.g., TRIAL FALL) pairs. There were 12 trials of each rhyming condition in each run. All words were monosyllabic, having neither homophones nor homographs, and were matched across conditions for written word frequency in children (Zeno et al., 1995) and the sum of their written bigram frequency (English Lexicon Project^1^). The stimuli had a mean age of acquisition of 6.6 (SD = 1.9) years (Kuperman et al., 2012) and we thus expected participants to be generally familiar with the experimental stimuli. We restricted our analyses to the two rhyming conditions (i.e., those associated with “yes” responses) to minimize language-irrelevant variance attributable to “yes” vs. “no” decision processing. Fixation trials (24 for each run) were included as a baseline and required the participant to press the “yes” button when a fixation-cross at the center of the screen turned from red to blue. Perceptual trials (12 for each run) were included in each run to permit contrasts against perceptual processing for a related study but were not used in the present study. These perceptual trials comprised two sequences containing tones (AA), or tones followed by non-alphabetic characters (AV). These stimuli were presented as increasing, decreasing or steady in pitch (for auditory stimuli) or height (for visual stimuli). Participants were required to indicate *via* button press whether the sequences matched (e.g., two rising sequences) or mismatched (e.g., a rising sequence followed by a falling sequence). The timing for the fixation and perceptual trials were the same as for the lexical trials. Each run had a different pseudo-randomly interleaved ordering of lexical, fixation and perceptual trials. The intertrial intervals varied between 2,200 and 2,800 and optimized for an event-related design using OptSeq^2^ to facilitate the modeling of overlapping hemodynamic responses. The lists were fixed across participants.

#### Functional MRI Data Acquisition

Participants were positioned in the MRI scanner with their head secured using foam pads and outfitted with an optical response box in the right hand. Visual stimuli were projected onto a rear-mounted screen viewed from a mirror attached to the inside of the head coil. Participants wore sound attenuating headphones to minimize the effects of the ambient scanner noise and deliver the auditory stimuli. Images were acquired using a 3.0 Tesla Siemens Trio scanner. A high resolution T1-weighted 3D structural image was first acquired for each subject (TR = 1,570 ms, TE = 3.36 ms, matrix size = 256 × 256, field of view = 240 mm, slice thickness = 1 mm, voxel size = 1 mm × 1 mm, number of slices = 160). Blood Oxygen Level Dependent (BOLD) functional images were acquired using a single-shot EPI (echo planar imaging) method, interleaved obliquely from bottom to top in a whole-brain acquisition (TE = 20 ms, flip angle = 80°, matrix size = 128 × 120, field of view = 220 × 206.25 mm, slice thickness = 3 mm (0.48 mm gap), number of slices = 32, TR = 2,000 ms, voxel size = 1.72 mm × 1.72 mm).

#### Functional MRI Data Preprocessing

fMRI data were analyzed using SPM8 (Statistical Parametric Mapping^3^). ArtRepair software^4^ was used during image preprocessing to correct for participant movement by replacing outlier volumes associated with interpolated values from the two adjacent non-outlier scans. Outlier scans were defined as those for which a signal change of more than 1.5% from the mean, or movement of more than one voxel along any axis was detected. No more than 10% of the volumes from each run and no more than four consecutive volumes were interpolated in this way. A single attempt was made to reacquire runs requiring replacement of more than 10% of the volumes or more than four consecutive volumes. Slice time correction was applied to minimize timing-errors between slices. Functional images were co-registered with the anatomical image and normalized to the Montreal Neurological Institute (MNI) ICBM152 T1 template.

#### Design and Analysis

A standard general linear model (GLM) analysis estimated the neural response associated with each experimental condition at each voxel within the brain by convolving the vector of event onsets for each of the trial types (four lexical, one perceptual and one fixation) with SPMs canonical hemodynamic response function (HRF), and using the convolved waveforms to predict the observed BOLD waveforms in each voxel. The goodness of fit between convolved and observed waveforms was thus computed as a standardized regression coefficient (beta) for each of the six trial types in a GLM predicting voxel-wise BOLD activation. These betas are conventionally used as a measure of the responsiveness of the neural populations within each voxel to each of the trial types.

A first-level GLM analysis was performed for each participant and included *t*-tests statistically contrasting the responsiveness of each voxel to the lexical conditions and to the fixation baseline (LEX > NULL). A second-level random-effects analysis of the single-subject (LEX > NULL) contrast followed, collapsing across all participants to verify that the pattern of fMRI activations for our sample was in-line with those reported in previous studies.

Our previous study had found differences between TD and RD children with respect to audiovisual integration in STG, FG, IPL and STS (McNorgan et al., 2013). We note that the group-level GLM analyses will show significant task-related activations in other brain areas, however, we did not include these additional regions in our region of interest (ROI) analysis. We took this approach primarily for two reasons: first, not all regions have theoretical ties to reading development or developmental reading disorders, making it difficult to interpret results associated with these regions. Second, though there may be significant task activations in a region, it does not follow that these activations will correlate with reading skills. Thus, because we applied a Bonferroni correction for multiple comparisons to our ROI analyses, including these regions would increase the Type II error rate with a diminishing likelihood of gaining novel theoretical insight. For these reasons, restricted our analyses to those reasons for which we had *a priori* hypothesis.

**Figure 2:**
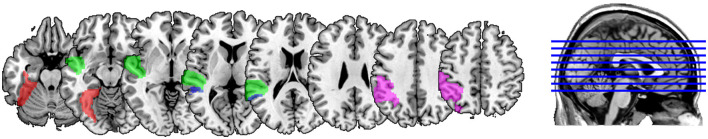
Anatomical extents of the atlas-based anatomical definitions of the masks used to constrain region of interest (ROI) definitions for fusiform gyrus (FG; red), superior temporal gyrus (STG; green), superior temporal sulcus (STS; blue) and inferior parietal lobule (IPL; magenta). Voxels falling within each of these regions that showed greater activity for lexical trials vs. baseline for a participant were included in that participant’s ROI for that anatomical label.

ROIs were generated for each participant, allowing us to identify and characterize the neural activations in atlas-based definitions of these regions taken from the Wake Forest University PickAtlas, which was also used to help identify peak activations in the GLM analysis^5^. Because the PickAtlas provides gyral but not sulcal definitions, 4 mm dilations of the STG and MTG PickAtlas regions were intersected to generate an atlas-based definition of the STS, from which the posterior-most third was taken as the pSTS, as we have done in our previously published studies exploring this region. The anatomical extents of these atlas-based definitions are illustrated in Figure 2. Participant-specific ROIs were generated separately for the AA and AV modalities (i.e., two sets of masks) by identifying within each of these anatomical regions those subsets of voxels showing numerically greater activation for all lexical conditions than for the fixation condition within that modality—that is, no participant’s ROIs included all the voxels included within these anatomical masks, but rather, these anatomical masks ensured that the functionally-defined ROIs for each individual were constrained to those anatomical regions prescribed by the hypotheses we were testing. An absolute statistical threshold was not applied because conventional statistical significance thresholds (e.g., *p* < 0.05) failed to select voxels in all regions for all participants, who notably come from a population for which reduced activation is commonly found among these regions (Richlan et al., 2009). These masks identified for each child voxels within these anatomical regions showing heightened activity under either unimodal AA and crossmodal AV task conditions, respectively. Importantly, because the same voxel selection criterion was used for all conditions and all participants, the ROI masks were bias-free.

We calculated mean signal strength across all rhyming lexical conditions and for the fixation baseline condition in each of the four regions separately for each task modality. Each calculation used the ROI mask for the corresponding task modality. Thus, for example, when computing the mean signal strength within the AA task data, the mean value for the FG was calculated overall FG voxels showing greater than baseline lexical activation in the AA condition, whereas, for AV task modality, this value was calculated overall FG voxels showing greater than baseline lexical activation in the AV condition. By calculating signal strength in this way, we avoided misleading comparisons between modalities that might arise from the assumption of similar spatial distributions of positive activations. Such an assumption could lead us to omit many relevant voxels or include many irrelevant voxels. Instead, we focused only and exactly on those voxels with any degree of positive association with the task for each modality condition.

Our ROI analysis submitted mean signal strength for the AA and AV task modality conditions and baseline signal strength and performance IQ to a hierarchical multiple regression with either raw Blending scores or raw Elision scores as the dependent measure of phonemic awareness. Because McNorgan et al. (2013) previously found no relation between audio-visual processing and Elision scores among these regions, our focus was in determining whether skill in the synthetic aspect of phonemic awareness might instead be predicted by unimodal processing, after accounting for variance predicted by nuisance regressors and by audio-visual processing. The sequence of regression steps forced age (in months), baseline signal and performance IQ in the first block as nuisance regressors, mean AV signal strength in the second block, and mean AA signal in the final block, predicting phonemic awareness as a function of the neural activity associated with both unimodal and crossmodal language processing. This approach conservatively controls for baseline signal strength, participant age, and performance IQ nuisance regressors but maximized sensitivity to any predictive ability of neural activity during the AV task. However, given the previously reported null effects for audiovisual processing in this population, we focus on whether unimodal processing during the AA task significantly predicts phonemic awareness after controlling for our nuisance predictors and audio-visual processing. The regression analyses were performed for each ROI, controlling for multiple comparisons, allowing us to determine whether task-related activity predicted synthetic phonemic awareness in each region. Because McNorgan et al. (2011) showed that dyslexic children demonstrate an atypical pattern of audiovisual integration-related processing, even under crossmodal conditions that should promote audiovisual integration, we predicted that phonemic awareness would be related to processing in the unimodal AA task condition, but not the crossmodal AV task condition, and only in regions associated with unimodal processing.

## Results

### GLM Analysis

**Figure 3:**
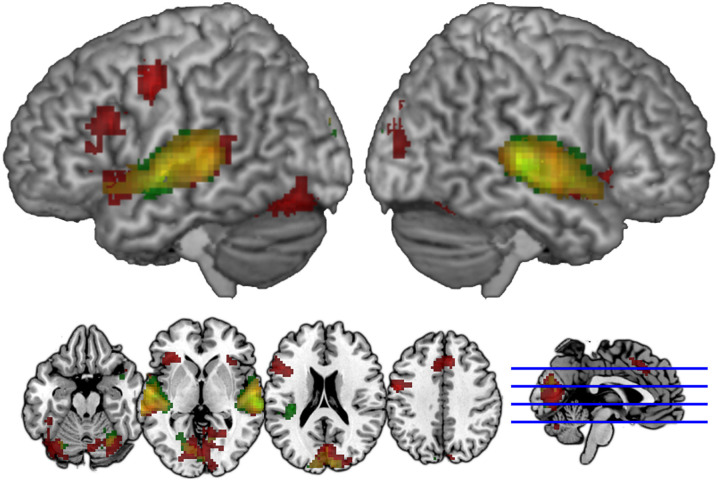
Spatial distribution of voxels demonstrating group-level lexicality effects (contrast of Lexical vs. Fixation trials) in the AV Crossmodal task (red) and AA Unimodal task (green). Overlapping modality effects appear in yellow. Clusters are extent-corrected at an FWE significance level of *p* < 0.05, with an uncorrected voxel-wise *p* < 0.001.

**Figure 4:**
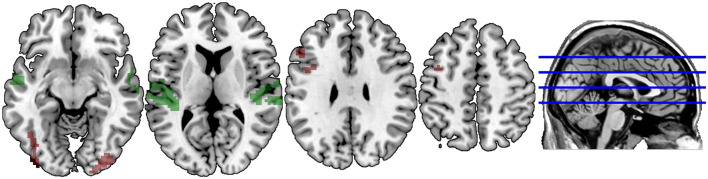
Spatial distribution of voxels demonstrating significantly greater lexicality effects in the Unimodal task (green) vs. the Crossmodal task, and demonstrating significantly greater lexicality effects in the Crossmodal task (red) vs. the Unimodal task. Clusters are extent-corrected at an FWE significance level of *p* < 0.05, with an uncorrected voxel-wise *p* < 0.001.

Figures 3, 4 illustrate significant clusters in the group-level GLM analysis using an uncorrected voxel-wise significance threshold of *p* < 0.001 and a cluster-level family-wise error rate of *p* < 0.05 (i.e., clusters of the obtained size are 5% likely to occur by chance under Gaussian random field theory). Focusing on the modality conditions in isolation (Figure 3), the analysis found both the unimodal AA and crossmodal AV task conditions were associated with activations significantly above baseline in a network of regions implicated in phonological processing (bilateral BA 21/22; STG) and visual/orthographic processing (BA 17/18/37; Cuneus, extending into FG and left calcarine fissure). Additionally, the crossmodal condition was associated with significant clusters in left IFG (BA 44/45; Broca’s area) and left precentral gyrus. Direct contrasts between the two task modality conditions (Figure 4) found the unimodal task was associated with significantly greater activation only in bilateral STG, whereas the crossmodal task was associated with significantly greater activation in bilateral occipitotemporal and left inferior frontal regions. The coordinates of peak maxima for these clusters are presented in Table 2. These overall results indicate that participants were engaging networks of regions commonly associated with phonological and orthographic processing of language, and did so under both task modality conditions. The task modality contrasts reflect the relative orthographic and phonological demands associated with each task modality: The unimodal task modality placed greater demand on bilateral primary and associative auditory processing regions, whereas the crossmodal task placed greater demand on bilateral visual processing regions and the left inferior frontal gyrus, which has been argued to play a critical role in visual word recognition (Cornelissen et al., 2009).

**Table 2:** Table of coordinates of peak activations for significant extent-corrected clusters of activation.

Unimodal						
Region	Size	pFWE	Max Z	*x*	*y*	*z*
R. Superior/Middle Temporal Gyrus (BA 22, 21)	861	<0.001	6.58	63	−27	3
L. Superior/Middle Temporal Gyrus (BA 22, 21)	1,050	<0.001	6.09	−57	−9	−3
R. Cuneus (BA 18)	515	<0.001	4.66	9	−90	24
R. Cerebellum	189	<0.001	4.51	27	−63	−24
**Crossmodal**						
R. Superior/Middle Temporal Gyrus (BA 22, 21)	703	<0.001	5.92	60	−12	3
L. Superior/Middle Temporal Gyrus (BA 22, 21)	1,009	<0.001	5.78	−54	−27	3
L. Precentral Gyrus (BA 6)	139	<0.001	5.15	−48	−3	42
R. Calcarine Sulcus (BA 17)	1,853	<0.001	5.05	3	−72	15
R. Middle Cingulum (BA 32)	239	<0.001	4.99	12	27	36
L. Inferior Frontal Gyrus (BA 44/45)	163	<0.001	4.85	−51	18	24
R. Insula (BA 13)	68	0.012	4.63	39	21	−9
**Crossmodal > Unimodal**						
L. Fusiform Gyrus (BA 37)	99	0.002	5.41	−42	−66	−15
L. Inferior Frontal Gyrus (BA 45)	147	<0.001	5.08	−45	18	24
R. Inferior Occipital Gyrus (BA 18)	91	0.002	4.15	33	−93	−9
**Unimodal > Crossmodal**						
R. Superior Temporal Gyrus (BA 22, 21)	349	<0.001	5.55	57	−18	0
L. Superior Temporal Gyrus (BA 22, 21)	431	<0.001	4.71	−57	−9	−3

*Note: L, left; R, right; BA, Brodmann Area (approx.); FDR, FDR-corrected significance level; Max, maximum. Size is measured in voxels. Coordinates reflect standard MNI space*.

### ROI Analysis

**Figure 5:**
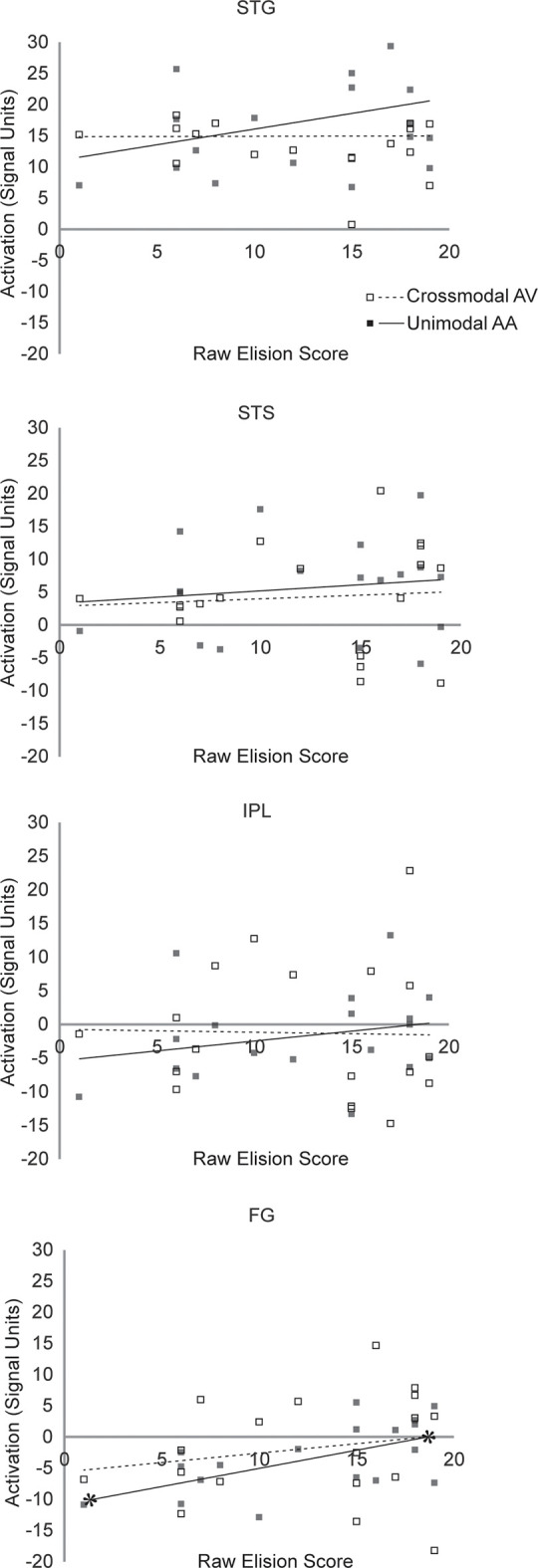
Scatterplot diagram of ROI activations as a function of Blending scores. Significant regression lines are capped with asterisks.

**Figure 6:**
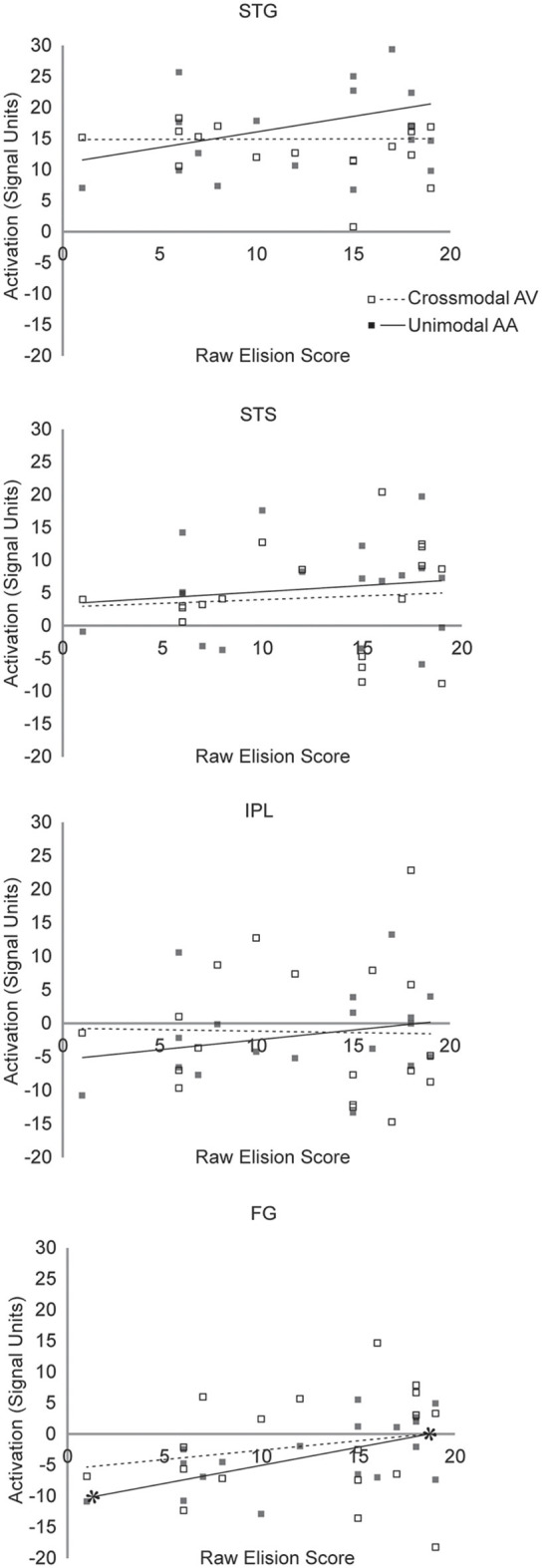
Scatterplot diagram of ROI activations as a function of Elision scores. Significant regression lines are capped with asterisks.

The exclusionary criteria for this study selected children clinically diagnosed with RD and no other cognitive or behavioral impairment. Consequently, though performance IQ and baseline fixation activity were included as nuisance regressors in the analyses that follow, it is unsurprising that neither were significant predictors in any of the regression analyses that follow, nor was age. Scatterplot diagrams for the regressions predicting Blending and Elision scores are presented in Figures 5, 6, respectively. All significance levels are reported using a Šidák family-wise error rate correction for multiple comparisons.

#### Superior Temporal Gyrus

The hierarchical linear regression found mean unimodal lexical task-related activity in the STG significantly improved prediction of raw Blending scores after accounting for all other regressor variables (*F*_(1,12)_ = 7.33, *p* = 0.038. In the final model, only unimodal activity was a significant predictor of raw Blending scores (*t*_(12)_ = 2.71, *p* = 0.019, partial *r*_(16)_ = 0.48, *f*^2^ = 0.96). Mean crossmodal lexical task-related activity in the STG was not a significant predictor of Blending scores (*t*_(12)_ = 0.641, *p* = 0.54). This pattern of results suggests that neural processing in STG is related to phoneme synthesis in children with RD under unimodal, but not crossmodal task conditions. The hierarchical linear regression found mean unimodal lexical task-related activity in the STG did not significantly improve the prediction of raw Elision scores after accounting for all other regressor variables (*F*_(1,12)_ = 2.887, *p* = 0.52. In the final model, neither mean unimodal activity (*t*_(12)_ = 1.699, *p* = 0.115) nor crossmodal activity in the STG (*t*_(12)_ = −0.279, *p* = 0.79) were significant predictors of raw Elision scores. This pattern of results suggests that neural processing in STG is unrelated to phonemic analysis ability in children with RD under either unimodal or crossmodal task conditions.

#### Fusiform Gyrus

The hierarchical linear regression found mean unimodal lexical task-related activity in the FG did not significantly improve the prediction of raw Blending scores after accounting for all other regressor variables (*F*_(1,12)_ = 0.193, *p* = 0.999. In the final model, neither mean unimodal activity (*t*_(12)_ = −0.439, *p* = 0.67) nor crossmodal activity in the FG (*t*_(12)_ = −0.497, *p* = 0.63) were significant predictors of raw Blending scores. This pattern of results suggests that neural processing in FG is unrelated to phoneme synthesis in children with RD under either unimodal or crossmodal task conditions. The hierarchical linear regression found mean unimodal lexical task-related activity in the FG marginally improved prediction of raw Elision scores after accounting for all other regressor variables (*F*_(1,12)_ = 7.51, but because we anticipated a null effect, this improvement was not significant after the familywise error rate correction was applied, *p* = 0.10. In the final model, mean unimodal activity (*t*_(12)_ = 2.739, *p* = 0.018, partial *r*_(16)_ = 0.59, *f*^2^ = 1.44) was a significant predictor of raw Elision scores, but crossmodal activity in the FG (*t*_(12)_ = 1.015, *p* = 0.22) was not. This pattern of results suggests that neural processing in FG may be weakly related to phoneme analysis ability in children with RD under unimodal but not crossmodal task conditions.

#### Superior Temporal Sulcus

The hierarchical linear regression found mean unimodal lexical task-related activity in the STS did not significantly improve the prediction of raw Blending scores after accounting for all other regressor variables (*F*_(1,12)_ = 2.109, *p* = 0.32. In the final model, neither mean unimodal activity (*t*_(12)_ = 1.452, *p* = 0.17) nor crossmodal activity in the STS (*t*_(12)_ = −0.889, *p* =* 0*.39) were significant predictors of raw Blending scores. This pattern of results suggests that neural processing in STS is unrelated to phoneme synthesis in children with RD under either unimodal or crossmodal task conditions. The hierarchical linear regression found mean unimodal lexical task-related activity in the STS did not significantly improve the prediction of raw Elision scores after accounting for all other regressor variables (*F*_(1,12)_ = 0.744, *p* = 0.96. In the final model, neither mean unimodal activity (*t*_(12)_ = 0.863, *p* = 0.41) nor crossmodal activity in the STS (*t*_(12)_ = −0.339, *p* = 0.74) were significant predictors of raw Elision scores. This pattern of results suggests that neural processing in STS is unrelated to phoneme analysis in children with RD under either unimodal or crossmodal task conditions.

#### Inferior Parietal Lobule

The hierarchical linear regression found mean unimodal lexical task-related activity in the IPL did not significantly improve the prediction of raw Blending scores after accounting for all other regressor variables (*F*_(1,12)_ = 0.09, *p* = 0.99. In the final model, neither mean unimodal activity (*t*_(12)_ = −0.304, *p* = 0.77) nor crossmodal activity in the IPL (*t*_(12)_ = −0.732, *p* = 0.48) were significant predictors of raw Blending scores. This pattern of results suggests that neural processing in IPL is unrelated to phoneme synthesis in children with RD under either unimodal or crossmodal task conditions.

The hierarchical linear regression found mean unimodal lexical task-related activity in the IPL did not significantly improve the prediction of raw Elision scores after accounting for all other regressor variables (*F*_(1,12)_ = 0.744, *p* = 0.93. In the final model, neither mean unimodal activity (*t*_(12)_ = 2.73, *p* = 0.36) nor crossmodal activity in the IPL (*t*_(12)_ = 1.015, *p* = 0.58) were significant predictors of raw Elision scores. This pattern of results suggests that neural processing in IPL is unrelated to phoneme analysis in children with RD under either unimodal or crossmodal task conditions.

## Discussion

The purpose of this study was to further investigate how online rhyme judgment under unimodal and crossmodal presentation conditions predicts a continuum of phonemic awareness ability in children with RD. We examined the relationship between measures of phoneme synthesis (Blending) and analysis (Elision) with a rhyme judgment task presented in unimodal auditory-only (AA) and crossmodal audiovisual (AV) presentations in a left hemisphere sub-network of reading regions including STG, pSTS, IPL, and FG. We predicted that children with RD would demonstrate a reliance on unimodal processing in unisensory regions, but would not show a similar reliance on crossmodal processing in known multisensory processing sites. Based on previous findings reported by McNorgan et al. (2013), we anticipated that Elision would be unrelated to neural processing throughout this network. The results indicate that, for children with RD, phoneme synthesis *via* blending phonemes into whole word representations is related to STG activity during unimodal rhyme judgment, and that this is a large effect that is likely to replicate. The results also suggested that phoneme analysis *via* the Elision task may be similarly modulated by unisensory regions in the FG during unimodal rhyme judgment, and, though this was also found to be a large effect, we interpret this finding cautiously. This pattern suggests that better phonemic awareness in children with RD is associated with unimodal phonological processing, and implies a reliance on unisensory rather than multisensory brain regions to resolve these phonemic awareness tasks.

These results extend previous literature regarding the relationship between crossmodal rhyme judgment and phonological awareness. McNorgan et al. (2013) found the reciprocal pattern of findings to those we describe here: TD, but not RD readers, demonstrated a significant relationship between crossmodal AV rhyming and phoneme analysis skills in multisensory brain areas. Those results demonstrated a disconnect between phoneme analysis in an Elision task and crossmodal congruency in RD readers. However, the null effect previously described emphasized brain processes present in TD readers that appeared to be absent in RD readers (McNorgan et al., 2013), providing only indirect insight into phoneme analysis in this population. The present study provides further insight into the dynamics of the neural processes in which lower-skill readers *do* apply phonemic awareness skills to online lexical processing. Specifically, lower-skill readers with higher phonemic awareness appear to engage unisensory processing regions to perform auditory rhyme judgments.

### Phonemic Awareness in Unisensory Brain Areas

Whereas reading is a quintessentially multisensory task—printed words are mapped to phonological representations, which, in turn, refine the orthographic system—it is important to bear in mind that the rhyming judgment task used here is phonologically-based. Synthesis of individual phoneme segments into a whole word relies heavily on phonological representation as well as phonological memory and retrieval in the STG (Simos et al., 2000; Temple et al., 2001; Turkeltaub et al., 2003). Phoneme synthesis is an early pre-literate skill that is predictive of reading ability (Perfetti et al., 1987; Ouellette and Haley, 2013). A straightforward interpretation of this result is that children with RD readily engage the phonological system involved in phoneme synthesis when making a phonological (rhyme) judgment. The features over which phonological similarity is evaluated may include not only basic acoustic information but also information likely to be tapped by phoneme synthesis (e.g., the sequence in which phonemes are combined). While these processes would not necessarily be facilitated by orthographic representation in the auditory-only condition in our task, converging evidence in TD readers shows that with increased reading skills the STG plays a greater role in visual letter processing (Blau et al., 2010). Fluent readers with stronger phonemic awareness skills show greater co-activation for print and speech processing in the STG (Frost et al., 2009; Preston et al., 2016). The present study adds to the body of evidence suggesting that RD is associated with less influence of visual input on phonological processing because processing under the AV presentation condition in STG—or any other ROI—was unrelated to any measure of phonological awareness.

Though the hierarchical regression failed to show that neural activity under the AA condition significantly improved prediction of phoneme analysis over AV neural activity and other nuisance regressors, the final model nonetheless indicated a significant relationship between phoneme analysis in the FG with unimodal lexical processing in the AA condition. We thus interpret this relationship cautiously, and in light of the large body of literature shows that RD is associated with under-activation of this region (for a review see McCandliss and Noble, 2003). Our finding does not contradict this research as this region was not activated above baseline for the auditory-only condition for the overall sample. Rather, this activation bore a linear relationship to the child’s phonemic awareness. Because we did not find a similar relationship in the AV condition, this indicates that orthographic processing in this region is driven by mechanisms other than phonemic awareness when visual orthographic input is available, and thus that phonemic awareness does not mediate the resolution of written words in children with RD. This is not to suggest that children with RD do not engage the orthographic system during reading; rather it would be consistent with a model of RD in which the orthographic system does not provide ongoing support in the resolution of phonology (McNorgan et al., 2014). This may be the optimal strategy if audiovisual integration processes fail to generate useful information from the orthographic representation.

We take the well-supported position that developmental reading difficulties arise in large part from an inability to accurately and quickly map between phonological and orthographic representations (Booth et al., 2004, 2007; Cao et al., 2006; Bitan et al., 2007). Multisensory interactions provide useful constraints on lexical activations in either modality to the extent that they help disambiguate multiple competing representations (Seidenberg and McClelland, 1989; Harm and Seidenberg, 1999). Indeed, Price and Devlin’s (2011) Interactive Account argues that the specialization of FG for orthographic processing is a consequence of internally-driven (i.e., top-down) phonological input facilitating the perceptually-driven (i.e., bottom-up) visual object processing system. This facilitation hinges on effective crossmodal integration. The introduction of additional disambiguating information helps reduce uncertainty and allows a clear winning lexical representation to emerge. It follows that ineffective crossmodal integration may provide no useful information, or even misleading information.

### Audiovisual Integration Underlies Mapping Between Orthography and Phonology

The results described above, along with the underactivation of the IPL and pSTS, indicate that the children with RD in our sample may not have effectively extracted the statistical regularities in the mapping between orthography and phonology. Children with RD may utilize more direct access to whole word representations for auditory rhyme judgment, rather than operating at a smaller grain size that would refine by higher phonemic analysis skills and more specialized orthographic processing (Ziegler and Goswami, 2005). As anticipated, we did not find a relationship between phoneme analysis and neural activity in multisensory integration sites, such as the pSTS and IPL. If multisensory integration within these regions is critical for mapping between phonological and orthographic representations, this pattern may explain the failure to find a relationship between phonemic awareness and audiovisual processing for our RD readers: continuity between the phonological, multisensory, and orthographic systems would imply that phonemic awareness is related to processing across all three systems. Because phonemic awareness is unrelated to processing in multisensory brain regions and in the crossmodal conditions, this suggests that the IPL or pSTS do not contribute towards mapping between orthographic and phonological representations in children with RD. Thus, though phonemic awareness may influence phonological and orthographic processing in children with RD, it does so without the coordination that audiovisual integration processes afford.

The results of the current study provide further support for the hypothesis that crossmodal integration between letters and speech sounds is impaired in children with RD. A large body of literature has shown a failure to integrate letters and speech sounds as a causal factor in dyslexia. Failure to integrate individual phonemes with graphemes in transparent orthographies such as Dutch and German has been documented in both event-related potential (ERP) and fMRI studies of children during literacy acquisition (van Atteveldt et al., 2009; Blau et al., 2010; Blomert, 2011; Bakos et al., 2017). However, a recent ERP study of English-speaking children challenged the letter-sound integration hypothesis (Nash et al., 2017). The authors found only mild deficits for letter-sound integration in RD children compared to age and reading skill matched children. One explanation of the apparent inconsistency between these findings and proponents of the letter-sound integration hypothesis is that dyslexia manifests differently in shallow and deep orthographies. Dyslexia in shallow orthographies, like Dutch, may be characterized by slow, effortful serial processing of letters, while in deep orthographies, such as English, the slow speed and effort may be at a larger grain size, such as the rhymes tested in our phonological task. The suggestion that orthographic depth likely interacts with RD is supported by a recent neuroimaging study that found distinct areas of under-activation in shallow and deep orthographies (Martin et al., 2016). However, in both types of orthographic systems, low skilled readers under-activate the occipitotemporal cortex. This under-activation of unimodal visual areas implies a reliance on access to unimodal representations, which may be degraded in RD. The results of the current study indicate that phonemic awareness, particularly phonemic analysis, is not active in the binding of orthographic and phonological representations for children with RD. The children with RD in the current study were near adolescent, spanning a range of reading ability, with some near-average performance on standardized tasks. Thus, the neurobiological profile outlined in this study signifies a persistence of deficits in mapping between modalities even after several years of reading instruction at school. This indicates that similar to visual word recognition, the neural processing engaged in phoneme synthesis and analysis relies on alternative mechanisms in children with RD.

### Limitations and Future Directions

The results of the current study suggest that both phoneme synthesis and analysis in children with RD rely on unisensory brain areas and unimodal processing. However, phonemic awareness as measured in the current study may be mediated by other factors such as attention, working memory, and overall language ability. The design of the between-subjects study was a within-subjects examination of how phonemic awareness skill is related to crossmodal processing. Future between-subjects experiments may utilize a broader range of phonemic awareness tasks (e.g., deletion, segmentation, letter rhyming) in addition to functional skills like reading fluency and comprehension. Experiments such as these, using appropriately matched groups, would support explorations of how phonemic awareness might differentially support reading development in RD and TD populations. Similarly, as the diagnostic labels associated with RD imply a heterogeneous range of deficits related to phonology, semantics, print processing, and general linguistic ability, larger studies may further examine individual differences in crossmodal lexical processing.

We constrained our ROIs to those regions that are proposed to have a specific role in the processing of orthography, phonology, or the integration of these elements, aligned with van Atteveldt et al.’s (2004) model of the left posterior integration network. Future studies may explore the relationship between right hemisphere structures and crossmodal processing of orthography and phonology in RD. Hemispheric differences between TD and RD readers are apparent, and may indicate compensatory mechanisms in RD in the right hemisphere (Pugh et al., 2001; Démonet et al., 2004; Shaywitz and Shaywitz, 2005). For phonological judgment tasks, children with RD demonstrate enhanced activity in the right compared to left inferior temporal gyrus (Corina et al., 2001). Activity in the right anterior insula and right STS are enhanced in adults with dyslexia compared to those without for audiovisual and visual lexical judgment, indicating that the right hemisphere recruitment of homologous structures occurs during crossmodal lexical processing (Kast et al., 2011). Examination of the right hemisphere recruitment related to phonological and crossmodal processing may further inform the understanding of how RD readers apply orthographic and phonological representations to lexical processing. A test of whether this compensatory recruitment varies between word and pseudoword trials may provide insight into whether these activations are driven by visual or semantic processing (or both) as argued by Pugh et al. (2001).

## Summary

We explored the relationship between phonemic awareness and modality presentation in children with RD along a continuum of reading ability. Previous fMRI studies have found that in TD readers, phonemic awareness skill is associated with crossmodal integration of phonology and orthography (Frost et al., 2009; McNorgan et al., 2013). For RD readers, we did not find any association between brain activation in crossmodal (AV) tasks and phonemic awareness. However, we did find significant brain-behavior correlations in the STG for the phonemic awareness measure of Blending with unimodal auditory-only presentation and in the FG with Elision with the unimodal auditory-only presentation. These significant brain-behavior correlations were found in unisensory areas implicated in the processing of orthography (FG) and phonology (STG). Using a hypothesis-driven, ROI-based approach, we did not find any significant correlations for pSTS and IPL, areas implicated in crossmodal integration across a number of domains, including language processing. Future studies may further examine the functional connectivity within this reading network to further elucidate how connectivity between these crossmodal regions are predictive of phonemic awareness in both high and low skilled readers.

Despite some of our RD participants having near-typical performance on standardized measures of phonemic awareness, as a group, our sample does not show a relationship between phonemic awareness and crossmodal integration in multisensory regions as found in TD children (McNorgan et al., 2013). Rather, the children with RD show a relationship between phonemic awareness and unimodal auditory processing in unisensory STG. This indicates that phonemic awareness remains related to phonological processing, but is not related to the integration of orthographic and phonological representations in RD readers, even after approximately 5–9 years of reading instruction at school. The educational implications of these results indicate that phonemic awareness skills, particularly performance in phoneme analysis is not reflective of advancing literacy skills in RD, and may rather be mediated by alternative strategies (Shaywitz et al., 2003). As such, educators and interventionists need to be careful in the interpretation of how phonemic awareness constructs relate to reading ability when designing instruction.

## Data Availability Statement

Data are available at Open Neuro (https://openneuro.org/datasets/ds001894/versions/1.3.0).

## Ethics Statement

This study was carried out in accordance with the recommendations of the Institutional Review Board at Northwestern University, with written informed consent from all subjects. All subjects gave written informed consent in accordance with the Declaration of Helsinki. The protocol was approved by the Institutional Review Board at Northwestern University.

## Author Contributions

MR, CM, and JB conceptualized the study. MR and CM were involved in data collection. CM and EG carried out the data analysis and created the figures. MR, CM, EG, and JB contributed to the drafting and editing of the final manuscript.

## Conflict of Interest

The authors declare that the research was conducted in the absence of any commercial or financial relationships that could be construed as a potential conflict of interest.

## Footnotes

^1^http://elexicon.wustl.edu

^2^http://surfer.nmr.mgh.harvard.edu/optseq

^3^https://www.fil.ion.ucl.ac.uk/spm/

^4^http://cibsr.stanford.edu/tools/human-brain-project/artrepair-software.html

^5^http://fmri.wfubmc.edu/software/pickatlas
