# Corrigendum: Children With Reading Difficulty Rely on Unimodal Neural Processing for Phonemic Awareness

**DOI:** 10.3389/fnhum.2020.00313

**Published:** 2020-08-21

**Authors:** Melissa Randazzo, Emma B. Greenspon, James R. Booth, Chris McNorgan

**Affiliations:** ^1^Department of Communication Sciences and Disorders, Adelphi University, Garden City, NY, United States; ^2^Department of Psychology, State University at Buffalo, New York, NY, United States; ^3^Department of Psychology, Monmouth University, New Jersey, NJ, United States; ^4^Department of Psychology and Human Development, Vanderbilt University, Tennessee, TN, United States

**Keywords:** reading difficulty, crossmodal integration, phonemic awareness, audiovisual integration, fMRI—functional magnetic resonance imaging, dyslexia

In the original article, there was a mistake in Figure 5 as published. The label for FG was replaced with STS. The corrected Figure 5 appears below.

**FIGURE 5:**
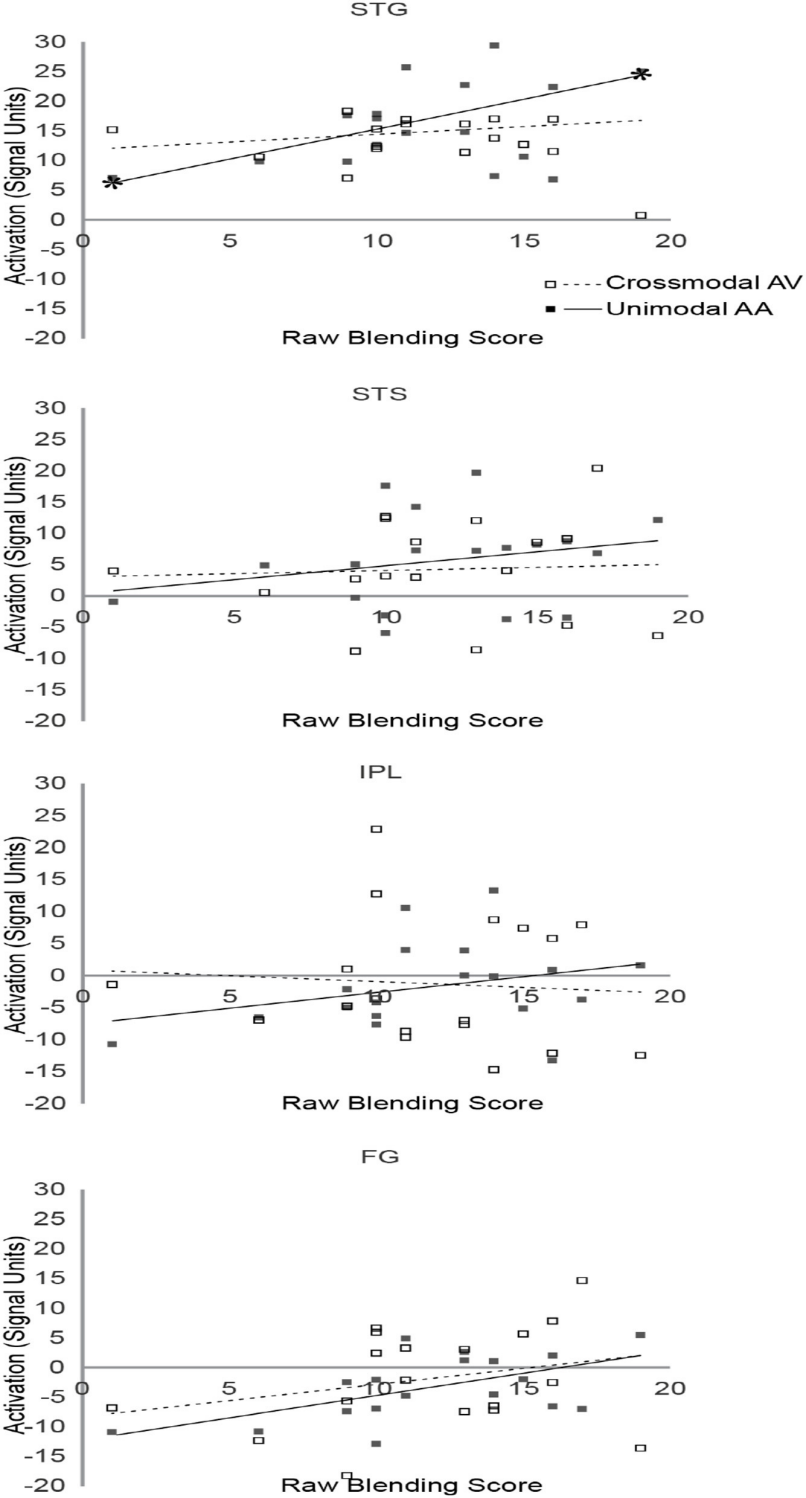
Scatterplot diagram of ROI activations as a function of Blending scores. Significant regression lines are capped with asterisks.

The authors apologize for this error and state that this does not change the scientific conclusions of the article in any way. The original article has been updated.

